# Illustration of extensive extracellular matrix at the epithelial-mesenchymal interface within the renal stem/progenitor cell niche

**DOI:** 10.1186/1472-6890-12-16

**Published:** 2012-09-25

**Authors:** Will W Minuth, Lucia Denk

**Affiliations:** 1 Department of Molecular and Cellular Anatomy, University of Regensburg, University Street 31, D - 93053, Regensburg, Germany

**Keywords:** Kidney, Interstitium, Stem/progenitor cell niche, Epithelial-mesenchymal interface, Extracellular matrix, Cupromeronic blue, Ruthenium red, Tannic acid, Electron microscopy

## Abstract

**Background:**

Stem/progenitor cells are promising candidates to treat diseased renal parenchyma. However, implanted stem/progenitor cells are exposed to a harmful atmosphere of degenerating parenchyma. To minimize hampering effects after an implantation investigations are in progress to administer these cells within an artificial polyester interstitum supporting survival. Learning from nature the renal stem/progenitor cell niche appears as a valuable model. At this site epithelial stem/progenitor cells within the collecting duct ampulla face mesenchymal stem/progenitor cells. Both cell types do not have close contact but are separated by a wide interstitium.

**Methods:**

To analyze extracellular matrix in this particular interstitium, special contrasting for transmission electron microscopy was performed. Kidneys of neonatal rabbits were fixed in solutions containing glutaraldehyde (GA) or in combination with cupromeronic blue, ruthenium red and tannic acid.

**Results:**

GA revealed a basal lamina at the ampulla and a bright but inconspicuously looking interstitial space. In contrast, GA containing cupromeronic blue exhibits numerous proteoglycan braces lining from the ampulla towards the interstitial space. GA containing ruthenium red or tannic acid demonstrates clouds of extracellular matrix protruding from the basal lamina of the ampulla to the surface of mesenchymal stem/progenitor cells.

**Conclusions:**

The actual data show that the interstitium between epithelial and mesenchymal stem/progenitor cells contains much more and up to date unknown extracellular matrix than earlier observed by classical GA fixation.

## Background

An increasing number of patients suffering from acute and chronic renal failure illustrates that other therapies than dialysis or transplantation have to be elaborated [1-5]. In consequence, the focus of actual research is directed to the implantation of stem/progenitor cells for the repair of diseased parenchyma [6-9]. Although this sounds simple, but a successful therapeutic protocol is rather difficult to perform due to the harmful environment in the diseased organ and the complex tasks that stem/progenitor cells have to fulfill during repair of renal parenchyma.

Implantation of stem/progenitor cells is normally started by an infusion via the blood vessel system or by an accidental injection into diseased renal parenchyme [10]. Once exposed to the harmful atmosphere stem/progenitor cells have to terminate the process of degeneration so that a successful repair of nephron structures can proceed [11,12]. However, critical review of actual literature shows that despite certain efforts a milestone in therapeutic success is up to date not in sight.

Regarding the complex processes during nephron repair it appears likely that an infusion or an accidental injection of stem/progenitor cells are not the ultimate methods to promote regeneration of parenchyma. As an alternative a new concept is favourized seeding stem/progenitor cells within a polyester fleece as an artificial niche and as a protective cover before an implantation under the organ capsule is made [13]. The strategy is to implant the cells at the earlier site of nephron formation for reactivation of this area [14-16].

Although the repopulation of an earlier stem/progenitor cell niche sounds simple, the biomedical performance is difficult to elaborate and needs intense research work. One of the basic problems is that only limited information is available about the creation of an artificial niche to keep implanted stem/progenitor cells in an environment maintaining competence for regeneration.

A reliable source for information may be contained in the renal stem/progenitor cell niche. During organ development nephrons arise in consecutive waves exclusively in the outer cortex of parenchyma. Astonishingly, the process of nephron induction proceeds always in a constant distance and close to the organ capsule [17]. In this particular embryonic zone the renal stem/progenitor cell niche is found. At this site epithelial stem/progenitor cells are localized within collecting duct (CD) ampulla branches originally derived from the ureteric bud. Cells within the tip of a CD ampulla communicate with the surrounding cap condensate containing nephrogenic mesenchymal stem/progenitor cells [18-22]. The intense reciprocal exchange of morphogenetic information including Pax2, Six1, Wnt9b, Ret, GDNF or BMP leads to a recruitment of only few mesenchymal stem/progenitor cells at the lateral edge of the cap condensate to form the pretubular aggregate [18,22,23]. For optimal development a special composition of extracellular matrix including related cell receptors maintains correct orientation of the CD ampulla to neighboring mesenchymal stem/progenitor cells [24]. First a comma- and then a S-shaped body arises as first visible morphological sign of nephron development [17,25-27].

It is unclear if the reciprocal exchange of morphogenetic factors during nephron induction occurs exclusively by diffusion or if also cell contacts are involved. Preventing uncontrolled dilution of morphogenetic information by diffusion one would assume that always a close contact is present between epithelial stem/progenitor cells within the tip of the CD ampulla and surrounding nephrogenic mesenchymal stem/progenitor cells [25,27]. However, the contrary is true. Immunohistochemical and morphological data have shown that around the tip of each CD ampulla an unique basal lamina and an interstitial space is established keeping nephrogenic mesenchymal cells in an astonishingly wide distance to neighboring epithelial stem/progenitor cells [28-30]. Light and electron microscopic analyses further show that after conventional fixation in glutaraldehyde the bright interstitial space does not exhibit recognizable extracellular matrix [31]. Furtheron, the striking interstitial space is not restricted to a single species, but was shown in developing rabbit [28,32], mouse [33-36], rat [37,38] and human [29,39] kidney.

The obvious separation of epithelial and mesenchymal cells within the renal stem/progenitor cell niche by a remarkable basal lamina and a wide interstitial space is conspicuous. Since in conventional fixation by glutaraldehyde this interstitial site does not exhibit recognizable extracellular matrix, it is assumed that masked molecules are contained as it is known for example from connective tissue [40]. Thus, the present investigation was performed to elaborate new structural features of the interstitium within the renal stem/progenitor cell niche. To detect new compounds of extracellular matrix in electron microscopy, fixation of tissue was performed with glutaraldehyde (GA) in combination with cupromeronic blue, ruthenium red and tannic acid. The currently applied fixation techniques illuminate that the interstitial interface between epithelial and mesenchymal stem/progenitor cells contains much more extracellular matrix as previously known.

## Methods

### Tissue preparation

One day old male and female New Zealand rabbits (Seidl, Oberndorf, Germany) were anesthetized with ether and killed by cervical dislocation. Both kidneys were immediately removed to process them for light and electron microscopy.

### Transmission electron microscopy

In the present investigation protocols of fixation were used developed years ago for the investigation of proteoglycans in cardiovascular structures [41] and extracellular matrix of mouse tectorial membrane matrix [42]. Without modifications the mentioned techniques were applied on embryonic parenchyma to visualize masked extracellular matrix within the renal stem/progenitor cell niche. In detail, specimens were fixed in following solutions for transmission electron microscopy:

1. Control series: 5% glutaraldehyde (GA, Serva, Heidelberg, Germany) buffered with 0.15 M sodium cacodylate, pH 7.4.

2. Experimental series with cupromeronic blue: 5% glutaraldehyde buffered with 0.15 M sodium cacodylate, pH 7.4. Then specimens were incubated in 0.1% cupromeronic blue (Santa Cruz, Heidelberg, Germany) and 0.1 M magnesium chloride hexahydrate (Sigma, Taufkirchen, Germany) dissolved in sodium acetate buffer pH 5.6. Counterstaining was performed with 0.5% sodium tungstate dehydrate (Sigma).

3. Experimental series with ruthenium red: 5% glutaraldehyde buffered with 0.15 M sodium cacodylate, pH 7.4 + 0.5% ruthenium red (Fluka, Taufkirchen, Germany).

4. Experimental series with tannic acid: 5% glutaraldehyde buffered with 0.15 M sodium cacodylate, pH 7.4 + 1% tannic acid (Sigma).

The period for fixation was for 1 day at room temperature. After several washes with 0.15 M sodium cacodylate the specimens were postfixed in the same buffer but containing 1% osmium tetroxide (Science Services, München, Germany). Then the tissue was washed with sodium cacodylate buffer and dehydrated in graded series of ethanols. Finally the specimens were embedded in Epon (Fluka), which was polymerized at 60°C for 48 h. Semithin and ultrathin sections were performed with a diamond knife on an ultramicrotome EM UC6 (Leica GmbH, Wetzlar, Germany). Sections were collected onto grids (200 mesh) and contrasted using 2% uranyl acetate and lead citrate as earlier described [31]. Sections were examined at 80 kV using an EM 902 transmission electron microscope (Zeiss, Oberkochen, Germany).

### Amount of analyzed specimens

A total of 58 exactly orientated renal stem cell niches was analyzed for the present study. All of the specimens were screened at least in triplicates. Performed experiments are in accordance with the Animal Ethics Committee, University of Regensburg, Regensburg, Germany.

### Definition of cells within the renal stem/progenitor cell niche

In the present paper the embryonic part of the developing rabbit kidney was described. For adaptation the nomenclature of previously published papers was used [20,31,43].

## Results

### Comparable view to the renal stem/progenitor cell niche

In the present experiment morphological features of the epithelial-mesenchymal interface within the renal stem/progenitor cell niche were analyzed. To obtain an always comparable view, it is essential to orientate a selected tissue block along the cortico-medullary axis of a lining collecting duct (CD) tubule. In consequence, all of the demonstrated micrographs show this perspective so that comparisons between different experimental series become possible. For clear recognition of the epithelial-mesenchymal interface the basal lamina at the tip of a CD ampulla is marked by a cross on each of the related micrographs.

### View by light microscopy

**Figure 1:**
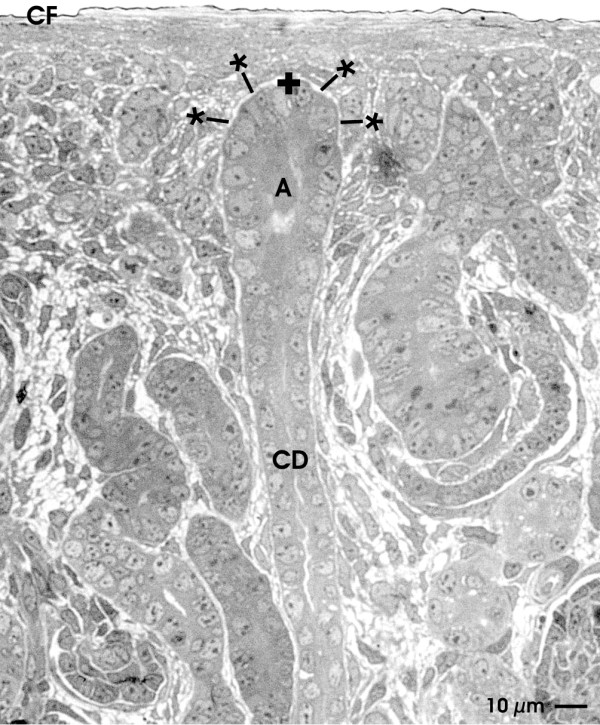
**Semithin section of the renal stem/progenitor cell niche underneath the organ capsule (CF).** Accurate orientation of parenchyma is obtained by sectioning in parallel to the lumen of a collecting duct (CD). Epithelial stem/progenitor cells are found within the tip of a ureteric bud derived CD ampulla (A). A thin layer of nephrogenic mesenchymal stem/progenitor cells is found between the tip of a CD ampulla and the organ capsule. Epithelial and mesenchymal stem/progenitor cells are separated by a wide interstitial space (lined asterisk). Coordinate for electron microscopy is labeled by a cross (**+**).

The epithelial-mesenchymal interface within the renal stem/progenitor cell niche can be visualized on a Richardson-labeled semithin section made from the outer cortex of the neonatal kidney (Figure 1). It is apparent that the tip of a CD ampulla containing epithelial stem/progenitor cells is found in an average distance of 20 μm underneath the organ capsule. Previous experiments revealed that this distance is maintained independently if a CD ampulla is in the process of branching or not [30]. Between the tip of a CD ampulla and the organ capsule a thin layer of mesenchymal stem/progenitor cells is present belonging to the cap condensate. Further the tip of the CD ampulla and surrounding mesenchymal stem/progenitor cells are not in close contact to each other but are separated by a clearly recognizable interstitial interface (Figure 1; lined asterisk).

### Transmission electron microscopy

**Figure 2:**
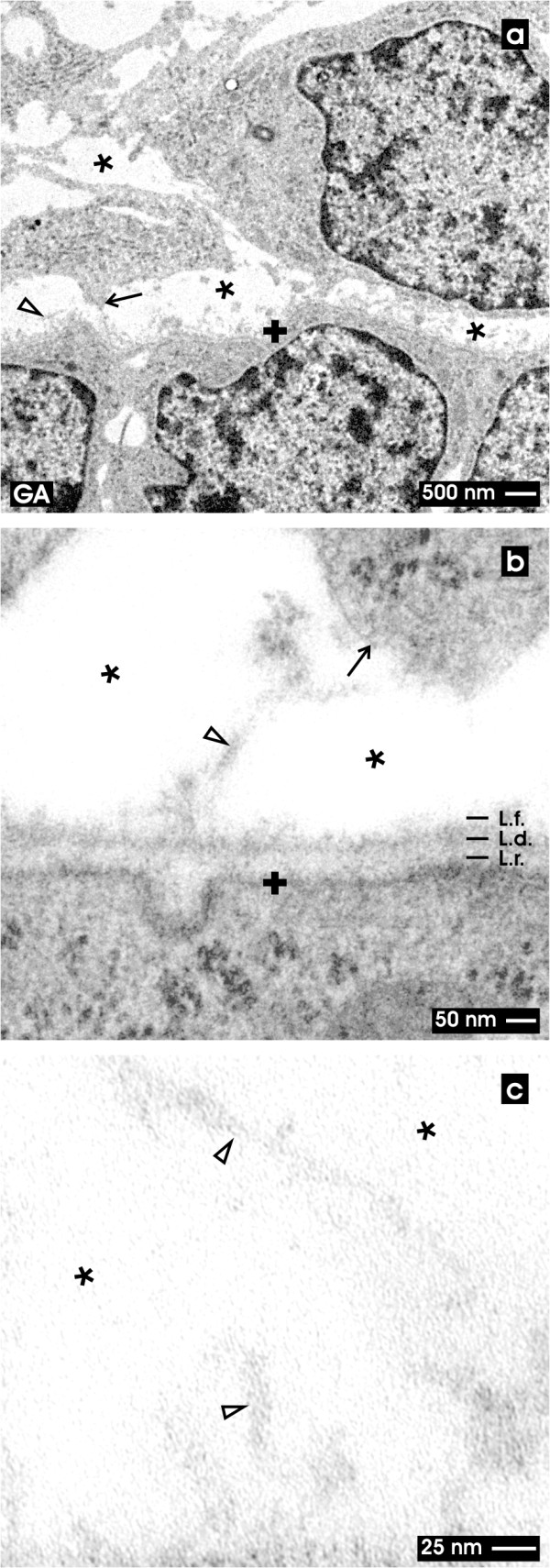
**TEM of the renal stem/progenitor cell niche after fixation in GA.** Low (a), higher (b) and high (c) magnifications illustrate the interstitial interface (asterisk) at the tip of a CD ampulla labeled by a cross (+). (**a**) Low magnification illustrates that epithelial stem/progenitor cells within the CD ampulla are separated from mesenchymal stem/progenitor cells by a consistently developed basal lamina and a bright interstitial space (asterisk). Protrusions from mesenchymal stem/progenitor cells line towards the CD ampulla (arrow). (**b**) Higher magnification shows that a basal lamina at the tip of the CD ampulla borders the interstitial interface. The basal lamina consists of a lamina rara (L.r.), lamina densa (L.d.) and lamina fibroreticularis (L.f.) with single fibers (clear arrow head) protruding to the interstitial space. (**c**) High magnification depicts the interstitial interface (asterisk) with single tiny fibers of extracellular matrix (clear arrow head). Other compounds cannot be recognized after staining with GA.

**Figure 3:**
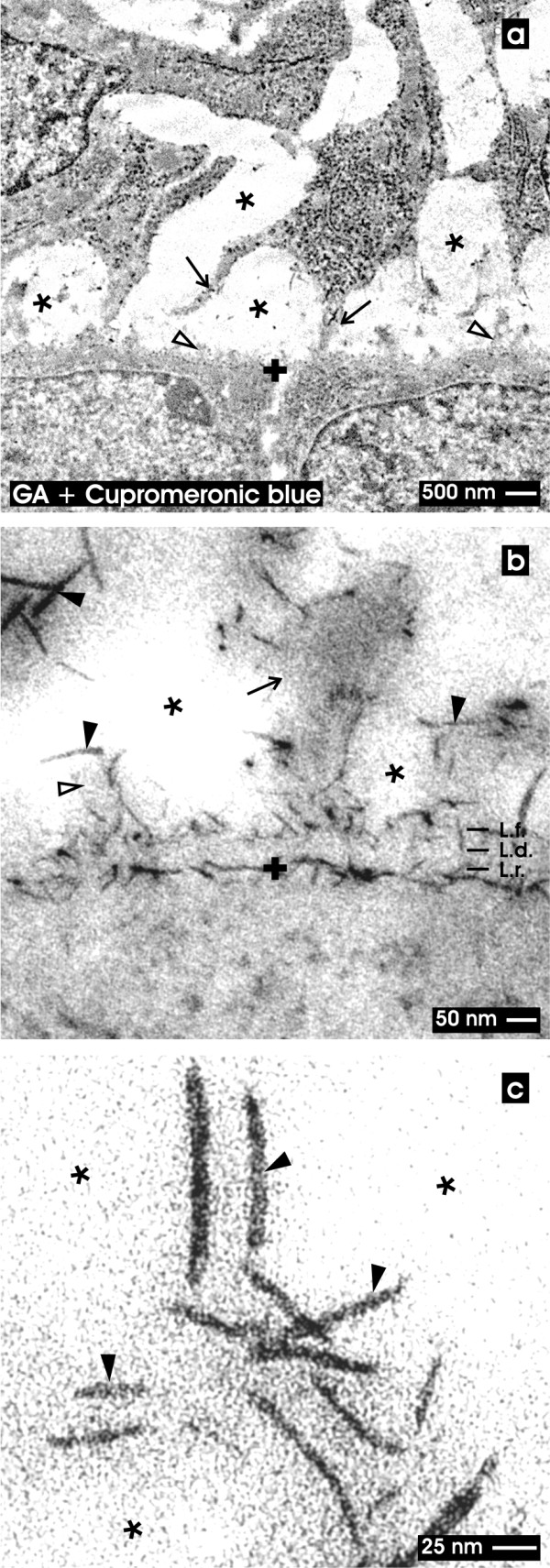
**TEM of the renal stem/progenitor cell niche after fixation in GA and cupromeronic blue. **Low (a), higher (b) and high (c) magnifications illustrate the interstitial interface (asterisk) at the tip of a CD ampulla labeled by a cross (+). (**a**) Low magnification depicts that numerous protrusions (arrow) from mesenchymal stem/progenitor cells contact the basal lamina at the tip of a CD ampulla covering epithelial stem/progenitor cells. The interstitial space between both kind of tissues appears bright (asterisk). (**b**) Higher magnification demonstrates that braces of proteoglycans line along the basal lamina of epithelial stem/progenitor cells within the CD ampulla. At the the lamina rara (L.r.) and lamina densa (L.d.) label is lacking, while along the plasma membrane and the lamina fibroreticularis (L.f.) numerous cupromeronic blue labeled molecules (filled arrow head) can be seen. The label is lining to adjacent cell protrusions of mesenchymal stem/progenitor cells (arrow). The interstitial interface appears bright. (**c**) High magnification demonstrates cupromeronic blue labeled proteoglycans (filled arrow head) at the bright interstitial interface (asterisk). Clear arrow head marks fibers at the lamina fibroreticularis.

**Figure 4:**
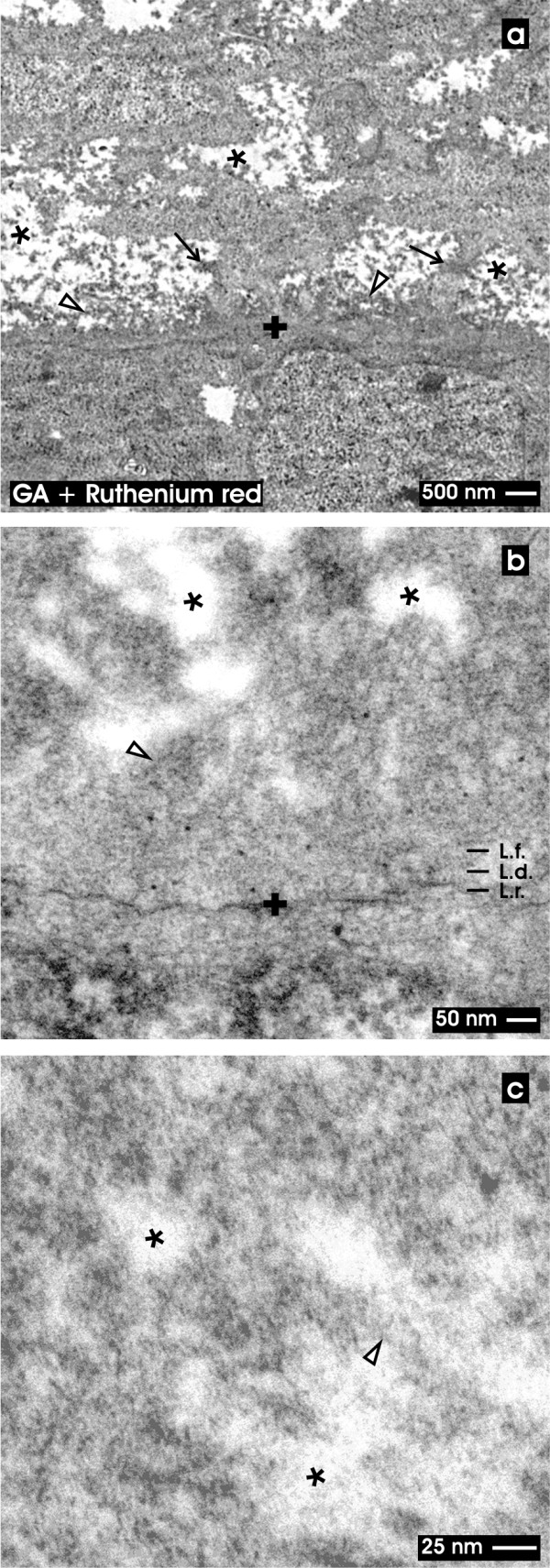
**TEM of the renal stem/progenitor cell niche after fixation in GA and ruthenium red.** Low (a), higher (b) and high (c) magnifications illustrate the interstitial interface (asterisk) at the tip of a CD ampulla labeled by a cross (+). (**a**) Low magnification shows a broad ruthenium red label lining along the basal lamina of the CD ampulla. Also neighboring mesenchymal stem/progenitor cells exhibit a punctuate label pattern at their surface. The cellular protrusions (arrow) show a coat labeled by ruthenium red and line through the interstitial space (asterisk) up to the lamina fibroreticularis (L.f.) of the CD ampulla. (**b**) Higher magnification illuminates that a three-laminar structure of the basal lamina of the CD ampulla cannot be seen. Instead numerous cloud-like bundles of ruthenium red labeled material are spanning from the outer surface of the CD ampulla through the interstitial space (asterisk) up to the surface of mesenchymal cells. (**c**) High magnification illustrates that the interstitial space (asterisk) is filled with clouds of extracellular material after ruthenium red staining. Clear arrow head marks fibers at the lamina fibroreticularis.

**Figure 5:**
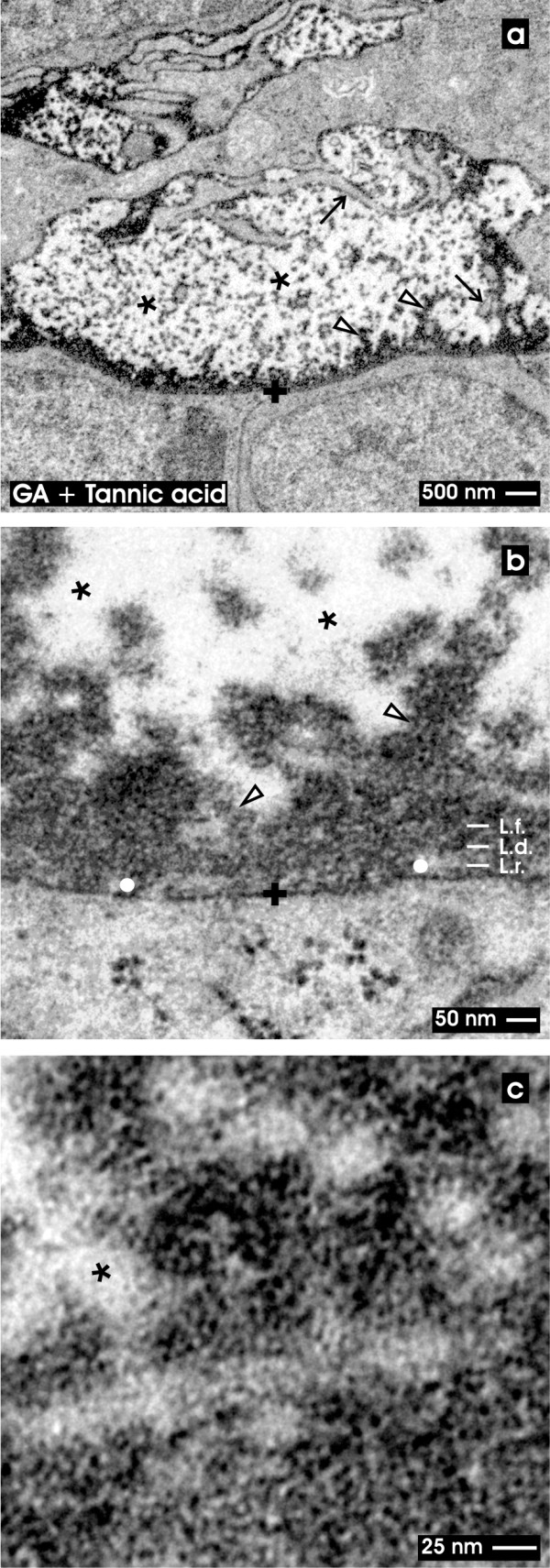
**TEM of the renal stem/progenitor cell niche after fixation in GA and tannic acid.** Low (a), higher (b) and high (c) magnifications illustrate the interstitial interface (asterisk) at the tip of a CD ampulla labeled by a cross (+). (**a**) Low magnification depicts that tannic acid covers as a dense coat the basal lamina of a CD ampulla. Further label lines along cellular protrusions of mesenchymal stem/progenitor cells (arrow). A punctuate pattern of tannic acid is found at the interstitial interface (asterisk). (**b**) Higher magnification illustrates a discontinuous label pattern at the lamina rara (L.r.; white dot), while the lamina densa (L.d.) and fibroreticularis (L.f.) depict an intense coat of tannic acid label. Further bundles of amorphous extracellular matrix are stained by tannic acid at the interstitial interface. (**c**) High magnification points out that label after tannic acid staining is not equally distributed but shows dense and less dense concentration. Clear arrow head marks fibers at the lamina fibroreticularis.

In the present experiments TEM was performed with embryonic renal parenchyma fixed by conventional glutaraldehyde (GA, Figure 2) or in combination with cupromeronic blue (Figure 3), ruthenium red (Figure 4) and tannic acid (Figure 5) to investigate extracellular matrix at the epithelial-mesenchymal interface within the renal stem/progenitor cell niche.

### Fixation with conventional GA

For control, in a first set of experiments specimens were fixed in a conventional solution containing GA (Figure 2). Low magnification shows that surrounding mesenchymal stem/progenitor cells keep distance and send out thin cellular protrusions towards the basal lamina of the CD ampulla (Figure 2a, arrow). The filigrane arrangement of cellular protrusions argues for an epithelial-mesenchymal interface that is well preserved by fixation. In so far the micrographs appear to reflect the natural situation and cannot be ascribed to an artifact due to fixation. It is obvious that the interstitium at the epithelial-mesenchymal interface appears bright and is free of amorphous or fibrous extracellular matrix.

Higher magnification in TEM shows that a consistently developed basal lamina covers epithelial stem/progenitor cells within the tip of the CD ampulla (Figure 2b). The basal lamina consists of a clearly visible lamina rara (L.r.), a lamina densa (L.d.) and a lamina fibroreticularis (L.f.). It can be observed that mesenchymal stem/progenitor cells send out protrusions to the surface of the CD ampulla.

Regarding low (Figure 2a), higher (Figure 2b) and high (Figure 2c) magnifications the interstitial space between the CD ampulla and the surrounding mesenchymal stem/progenitor cells appears bright and is free of extracellular matrix. Only single and faint fibers of extracellular matrix are lining from the tip of the CD ampulla through the wide interstitial space towards mesenchymal stem/progenitor cells (Figure 2b, clear arrow head).

### Fixation with GA and cupromeronic blue

In the second series solution with GA containing cupromeronic blue was applied for fixation (Figure 3). Low magnification illustrates the basal side of epithelial stem/progenitor cells within the tip of the CD ampulla (Figure 3a). It is obvious that the typical appearance of the basal lamina covering the tip of a CD ampulla yet is not visible. Mesenchymal stem/progenitor cells stay in distance to the CD ampulla and send out long protrusions contacting the basal lamina at the tip of a CD ampulla.

Higher magnification in TEM reveals that the basal lamina of the CD ampulla does not exhibit a clearly recognizable lamina rara (L.r.), lamina densa (L.d.) and lamina fibroreticularis (L.f.) (Figure 3b). However, cupromeronic blue treatment exhibits label along the basal plasma membrane and lamina fibroreticularis, while label within the lamina rara and lamina densa cannot be recognized. In longitudinal and vertical view of cupromeronic blue labeled specimens it can be seen that cellular protrusions from mesenchymal stem/progenitor cells span through the interstitial space to contact the lamina fibroreticularis at the tip of the CD ampulla. However, length and density of cupromeronic blue labeled proteoglycan braces differ significantly. At the surface of cellular protrusions labeled molecules exhibit a length of 100 nm, while within the basal lamina of the CD ampulla molecular braces with 50 nm are detected.

High magnification demonstrates proteoglycans contrasted by cupromeronic blue at the outer side of a CD ampulla and on protrusions of mesenchymal stem/progenitor cells (Figure 3c, filled arrow head).

### Fixation with GA and ruthenium red

In the third series of experiments specimens were fixed in GA including ruthenium red (Figure 4). Under low magnification in TEM it can be seen that the basal lamina of the CD ampulla contacting the interstitial space appears completely different as compared to previous series. The typical three-laminar structure of the basal lamina detected after classical GA fixation (Figure 2a) is not any more visible after ruthenium red label (Figure 4a). Instead a ribbon of intensive ruthenium red marker surrounds the basal aspect of the CD ampulla. Further cellular protrusions of mesenchymal stem/progenitor cells exhibit an excessive and roughly punctuate pattern on their surface. It can be recognized that individual cellular protrusions line through the interstitial space up to the lamina fibroreticularis at the tip of the CD ampulla.

Higher magnification in TEM of ruthenium red labeled specimens depicts that the basal lamina at the tip of the CD ampulla does not exhibit a recognizable lamina rara (L.r.), lamina densa (L.d.) and lamina fibroreticularis (L.f.) (Figure 4b). Instead the known layers of the basal lamina are comprised as a common broad ribbon covering the complete tip of the CD ampulla. From the area of the lamina fibroreticularis strands of extracellular matrix line into the interstitial space.

In addition, bundles of translucent fibers become visible within the interstitial space. Their center appears translucent, while the surface is covered by extracellular matrix marked by intense ruthenium red label. Since the fibers do not exhibit a repeating period, they cannot be ascribed to a certain type of collagen. It is further visible that the neighboring mesenchymal stem/progenitor cells are covered by a roughly structured coat labeled by ruthenium red.

High magnification in TEM depicts that ruthenium red label is not only on the surface of cells but is also found in form of extended clouds on neighboring extracellular matrix within the interstitial space (Figure 4c).

### Fixation with GA and tannic acid

In the last series fixation was performed by GA and tannic acid. Low magnification focuses to the basal aspect at the tip of a CD ampulla (Figure 5a). The micrograph clearly depicts that the complete basal lamina is covered by an electron-dense coat as detected after fixation with GA containing ruthenium red (Figure 4a). The intensively stained pattern protrudes from the basal lamina of the CD ampulla through the interstitial space towards the surface of neighboring mesenchymal stem/progenitor cells.

Higher magnification in TEM illuminates that intense tannic acid label is found at the basal lamina covering the tip of the CD ampulla (Figure 5b). However, only a discontinuously labeled lamina rara (L.r.) becomes visible, while the lamina densa (L.d.) and lamina fibroreticularis (L.f.) are seen as a broad ribbon. Further tannic acid labels to a high degree strands of extracellular matrix within the interstitial space. All protrusions and the cell surface of neighboring mesenchymal stem/progenitor cells exhibit an intense coat of tannic acid positive material. It is obvious that not the complete interstitial space but only part of it is labeled by tannic acid. In so far the result speaks in favour for a stain-specific label and not for an unspecific background signal.

High magnification in TEM finally demonstrates that tannic acid label is not equally distributed but is concentrated in particular areas of the interstitial space (Figure 4c).

In conclusion, light microscopy (Figure 1) and TEM (Figures 2, 3, 4 and 5) depict that epithelial stem/progenitor cells within the CD ampulla and the surrounding mesenchymal stem/progenitor cells are separated by an astonishingly structured interstitial space. Mesenchymal stem/progenitor cells send out long protrusions into the interstitial space to contact the lamina fibroreticularis covering the tip of a CD ampulla. In addition, fixation of tissue in conventional GA shows a clear but unspectacularly appearing interface between epithelial and mesenchymal stem/progenitor cells (Figure 2).

In contrast, applying advanced fixation with GA in combination with cupromeronic blue (Figure 3), ruthenium red (Figure 4) or tannic acid (Figure 5) illustrates that the interstitial space contains an unexpected amount of up to date not identified extracellular matrix. It is most astonishingly that the extracellular matrix is not restricted to the lamina fibroreticularis but widely extends through the interstitial space to reach protrusions and the body of neighboring mesenchymal stem/progenitor cells.

## Discussion and conclusions

In the kidney the extracellular matrix consists on the one hand of collagen type IV, laminins, nidogens and proteoglycans found within the basal lamina of contained epithelial structures [44] and on the other hand of interstitial proteins such as collagen type III sustaining as endoskeleton the three-dimensional structure of parenchyma [45,46]. In the complementary space fluid is crossing between collagen fibers, tubules and blood vessels to provide the parenchyma with nutrition, hormones, morphogenetic factors and respiratory gas [47-49]. Both extracellular matrix and complementary fluid space is known as interstitium.

A special meaning has the interstitium during development of the kidney. Numerous reciprocal morphogenetic interactions within the renal stem/progenitor cell niche control the development of nephrons and the spatial organization of parenchyma at the right site and at the right time [25,50]. In detail, surprisingly little knowledge is available about the molecular composition of this interstitial interface. At this unique site epithelial stem/progenitor cells within the tip of a ureteric bud derived CD ampulla are separated from surrounding nephrogenic mesenchymal stem/progenitor cells by an individual concentration of cellular anchorage proteins and related extracellular matrix [24,31,51]. Astonishingly, during nephron induction morphogenetic factors have to cross this layer of extracellular matrix. However, up to date it is an unsolved question if reciprocal exchange of morphogenetic information occurs exclusively via free diffusion through this interstitial interface or if also factors are involved bound on extracellular matrix. Another question in this coherence is whether and to what extend cellular contacts between epithelial and mesenchymal stem/progenitor cells are involved in the exchange of morphogenetic information [52].

When diffusion of factors is assumed during the process of nephron induction, one would expect a close contact between interacting cells so that uncontrolled dilution of morphogenetic information is prevented. In contrast, previous and present experiments demonstrate that after conventional fixation by GA an astonishingly wide interstitial space separates epithelial and mesenchymal stem/progenitor cells (Figures 1, 2; asterisk) [28,31,43,53]. Further it was shown that numerous cellular protrusions from mesenchymal stem/progenitor cells are lining through the interstitial space to contact the lamina fibroreticularis at the tip of a CD ampulla (Figures 2a, 3a, 4a, 5a; arrow). TEM further depicts that morphology and orientation of cellular protrusions looks fully intact indicating that the interstitial space including filigree protrusions of mesenchymal stem/progenitor cells appears real and is not caused by a fixation artifact.

The present data clearly demonstrate that conventional fixation with GA does not illuminate all of the structural compounds contained in the interstitial interface of the renal stem/progenitor cell niche (Figure 2b). Actual data further show that alterations of the fixation protocol by addition of cupromeronic blue (Figure 3b), ruthenium red (Figure 4b) and tannic acid (Figure 5b) exhibit structures in the interstitium, which are not earlier observed by classical fixation with GA (Figure 2b). For example, fixation in GA including cupromeronic blue illuminates a coat of earlier not known proteoglycan braces at the basal lamina at the tip of the CD ampulla. These fibrillar molecules are contained in the basal plasma membrane, do not occur in the lamina rara and lamina densa, but are frequently distributed within the lamina fibroreticularis (Figure 3b). Most interestingly, when protrusions from mesenchymal stem/progenitor cells contact the lamina fibroreticularis, cupromeronic blue labeled fibrillar molecules envelop them like a sock (Figure 3c).

Further fixation of specimens in GA containing ruthenium red (Figure 4) or tannic acid (Figure 5) depicts that the interstitial interface within the renal stem/progenitor cell niche contains an unexpectedly high amount of amorphous extracellular matrix. Material contrasted by ruthenium red (Figure 4b) and tannic acid (Figure 5b) is strongly associated to all three layers of the basal lamina at the tip of the CD ampulla. In addition, the labeled material is lining from the lamina fibroreticularis in form of striking bundles through the interstitial space up to the surface of mesenchymal stem/progenitor cells.

**Figure 6:**
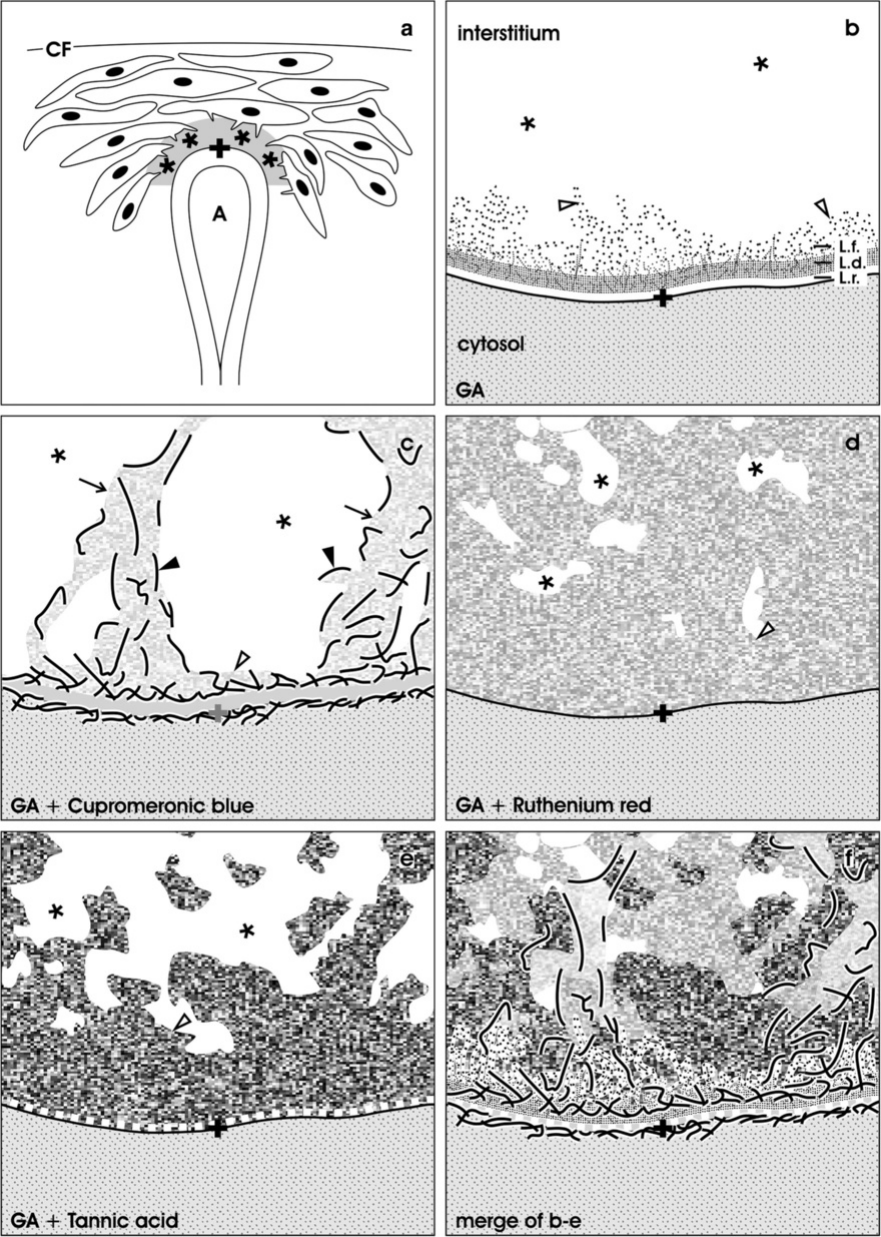
**Illustration of detected structures at the interstitial interface within the renal stem/progenitor cell niche.** (**a**) Mesenchymal stem/progenitor cells are separated from the epithelial stem/progenitor cells within the CD ampulla (A) by an extended interstitial interface (asterisk). Capsula fibrosa (CF). (**b**) After fixation with GA the interstitial interface (asterisk) is limited by the basal lamina of the CD ampulla. It consists of a lamina rara, lamina densa and lamina fibroreticularis. (**c**) After fixation in GA containing cupromeronic blue proteoglycans can be recognized lining along the basal lamina of the CD ampulla and protrusions of mesenchymal stem/progenitor cells. (**d**) Fixation with GA and ruthenium red shows a dense band along the basal lamina of the CD ampulla and numerous clouds of amorphous extracellular matrix within the interstitial space (asterisk). (**e**) Fixation with GA and tannic acid exhibits label at the basal lamina of the CD ampulla and bundles of extracellular matrix in the interstial interface (asterisk). (**f**) Merge of information found in illustrations **b-e**. It can be recognized that fixation with GA (**b**) does not depict extracellular matrix within the interstitium of the renal stem/progenitor cell niche. Label with cupromeronic blue (**c**), ruthenium red (**d**) and tannic acid (**e**) illustrates that the interstitial interface contains extracellular matrix. Cupromeronic blue (**c**), ruthenium red (**d**) and tannic acid (**e**) do not label the same but different kinds of molecules. Clear arrow head marks fibers at the lamina fibroreticularis. Arrow labels protrusions of mesenchymal stem/progenitor cells, filled arrow head illustrates labeled proteoglycans

Finally, TEM and schematic illustrations demonstrate that the extracellular matrix contrasted by cupromeronic blue (Figures 3, 6c) ruthenium red (Figures 4, 6d) or tannic acid (Figures 5, 6e) is connecting to an unexpectedly high degree both epithelial and mesenchymal stem/progenitor cells, while conventional fixation with GA (Figures 2, 6b) does not show this striking feature. The complementary space between the ruthenium red and tannic acid-positive material is free of any recognizable structures. It appears that this bright space non-labeled by cupromeronic blue, ruthenium red or tannic acid is the compartment, where interstitial fluid is crossing.

Thus, the present investigation illustrates that the interstitial interface of the renal stem/progenitor cell niche shows after fixation in GA containing cupromeronic blue (Figure 6c), ruthenium red (Figure 6d) and tannic acid (Figure 6e) more and different extracellular matrix as earlier demonstrated by conventional fixation by GA (Figure 6b). Experiments are under work to elaborate the molecular composition and physiological tasks of the detected extracellular matrix (Figure 6f). In each case its wide distribution and function must be reconsidered, since free diffusion of morphogenetic molecules is not promoted but appears to be restricted.

## Competing interests

The authors declare that they have no competing interests.

## Pre-publication history

The pre-publication history for this paper can be accessed here:

http://www.biomedcentral.com/1472-6890/12/16/prepub
